# A change of heart: a transforming view of cardiac function

**DOI:** 10.1038/s41536-017-0030-3

**Published:** 2017-09-04

**Authors:** Alexander R. Pinto

**Affiliations:** 1 Australian Regenerative Medicine Institute, Monash University, Melbourne, VIC Australia; 2 The Jackson Laboratory, Bar Harbor, ME USA

The heart is a landscape made up of complex and interactive networks of cells. Recently we have started to envision and explore this landscape in much sharper resolution. A new picture of cardiac physiology has emerged as studies mapping the cellular landscape of the heart have transformed our understanding of fundamental concepts of cardiac function, such as how the heart beats. While these discoveries have broad implications for basic cardiac biology and cardiology, they are particularly pertinent for cardiac regenerative medicine.

**Fig. 1:**
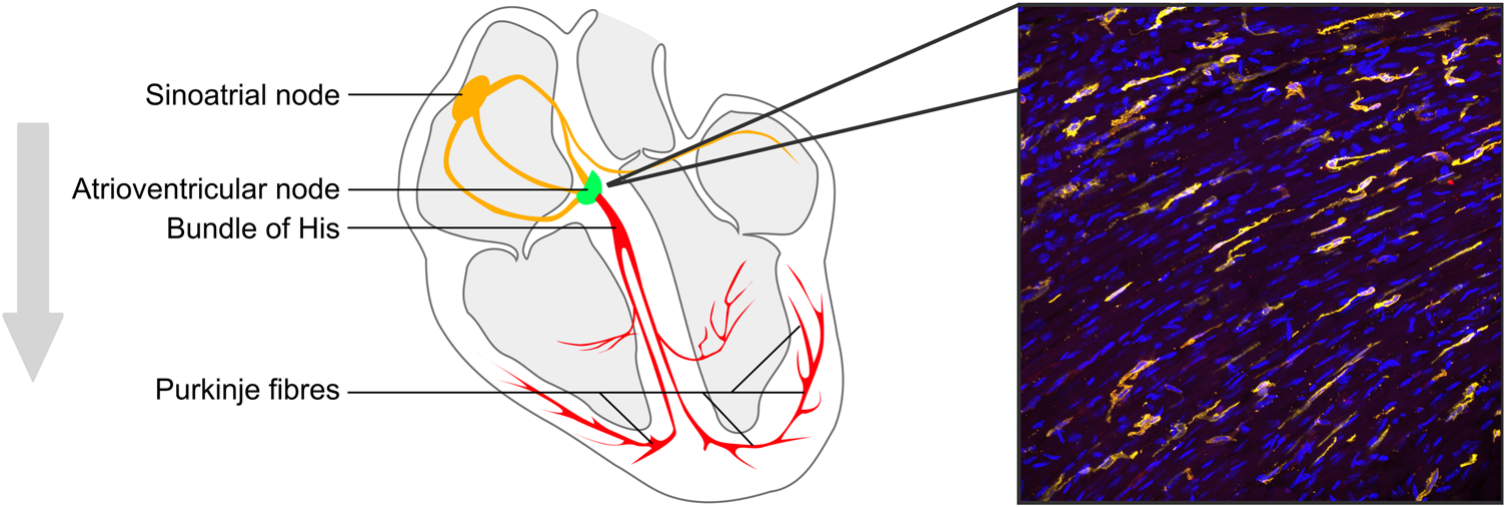
Cardiac conductance regulated by macrophages. Simplified schematic of the cardiac conductance pathway. *Gray arrow* indicates order of depolarization events leading to cardiac contraction. Micrograph shows nuclei (*blue*) and cardiac macrophages (*yellow*), which are highly enriched at the atrioventricular (AV node). Macrophages modulate AV node function and depletion of macrophages leads to disruption of AV node function (AV block) and arrhythmia

The rhythmic beating of the heart involves a coordinated series of events originating in, and propagated by, distinct cardiac cells at specific loci (Fig. 1). The beginning of a beat originates in the right atrium at the sinoatrial node (SAN)—the principal cardiac pacemaker. The SAN initiates an electrical impulse stimulating the contraction of atria and transmitting impulses to the atrioventricular node (AVN), which coordinates subsequent signals. The AVN signals travel through the interventricular septum via the bundle of His which in turn relays them to Purkinje fibers of the ventricles, causing contraction of ventricular muscles.

It has been the prevailing view that specialized cardiomyocytes at the SAN and AVN coordinate these electrical pacemaker functions. However recent findings by Narhrendorf and colleagues (2017)^1^ have demonstrated that the cardiac macrophage, a cell type of the innate immune system, is integral for normal rhythmic beating of the heart, challenging our previous thinking.

To demonstrate this surprising role of immune cells in cardiac conductance, the authors applied a range of genetic and imaging approaches. Using genetic reporters and immunohistochemistry, they showed that macrophages are highly enriched at the AVN in mice and humans. Next they demonstrated that macrophages contact cardiomyocytes at the AVN via the gap junction protein Connexin 43 (Cx43). Cx43 forms punctate junctions between macrophages and cardiomyocytes, resulting in the joining of cytosolic compartments and electrical coupling of the two cell types. By employing optogenetics, through which cells can be electronically activated by impulses of light, the authors showed that depolarization of macrophages improves AVN conductance.

The study also highlighted how important macrophage-cardiomyocyte interaction is for cardiac rhythm. Macrophage-specific genetic ablation of Cx43 delayed AVN conductance. More strikingly, depletion of macrophages altogether resulted in AVN block, where transmission of impulses from the AVN was delayed or blocked completely, resulting in arrhythmia.

These remarkable findings offer a new perspective on the role of macrophages in the heart and other tissues more broadly. Macrophages have been classically conceptualized as housekeeping cells which clear cell debris and act as sentinels for tissue damage and foreign particles. But another recent report demonstrated that paracrine signaling by macrophages affects cardiac conductance, in an IL-1β dependent manner.^2^ These studies further complicate our understanding of macrophage biology, and underscore the importance of macrophages in the heart beyond tissue maintenance.

While the role of macrophages in cardiac healing^3^ and neonatal heart regeneration^4^ has been characterized, little has been known of the functions of resident cardiac macrophages. Indeed, it is 5 years since a significant resident population of macrophages was first identified in the heart and systematically analyzed.^5^ This initial research found that cardiac macrophages have distinct characteristics compared to other tissue macrophages, including a robust inflammation-dampening phenotype. Building on this work, much more has been discovered, including that cardiac macrophages develop from cells that seed the heart early in embryonic development^6, 7^ with contributions from circulating monocytes.^6–8^ Further studies have also shown that macrophages are a complex cell population^6–9^ with both their heterogeneity and function altering with development^6, 9^ and aging.^9^ However, until now, there was no clear evidence macrophages were integral to rudimentary cardiac functions.

While we are gaining a more sophisticated understanding of the role of macrophages in the heart, what about other non-myocytes? The long-held dogma that the heart is principally comprised of cardiomyocytes and fibroblasts has also been challenged. Recent work has demonstrated that this concept is incorrect and that the most abundant non-myocyte cell type in humans and mice is the endothelial cell, which outnumbers the cardiomyocyte.^10^ Fibroblasts, which were previously considered the most abundant non-myocyte, are far less prevalent (~15–20% of non-myocytes). Cardiac non-myocytes are much greater in number than cardiomyocytes, and in addition to macrophages, many non-myocyte cell types express Cx43 and contribute to conductance and rhythm in the uninjured and injured heart.^11, 12^ Indeed, endothelial cell ablation of Cx43 is reported to induce bradycardia,^13^ and fibroblasts too electrically couple with cardiomyocytes.^14^ Therefore, it is plausible that multiple cell types contribute to cardiac conductance.

Our transforming view of cardiac cell biology should inform development of therapeutic strategies for cardiac disease. Cardiac non-myocytes such as macrophages have the capacity to affect all areas of cardiac physiology and likely are associated with the etiology of more cardiac diseases than previously appreciated. This may be particularly true for idiopathic cardiac syndromes, where macrophages and other non-myocytes have not been examined in detail. Replenishment of key non-myocyte cell types is also likely required to fully restore heart function after injury, in addition to cardiomyocytes. Studies concerned with cardiac regeneration should, therefore, take into account the full range of cell types found in the heart and their role in cardiac function.
